# Influence of Bcl-1 gene polymorphism of glucocortucoid receptor on phenotypic expressions of bronchial asthma

**DOI:** 10.1186/2045-7022-5-S2-P10

**Published:** 2015-03-23

**Authors:** Vladyslava Kmyta, Lyudmyla Prystupa

**Affiliations:** 1 Sumy State University, Sumy, Ukraine; 2 Sumy State University, Internal Medicine department postgraduate educatio, Sumy, Ukraine

## 

Bronchial asthma (BA) is a chronic polyetiologic disease, which is determined by many environmental and genetic factors. The clinical picture occurs as a result of complex interactions among genes, and mutual influence of a genotype and environment upon each other. One of the genes involved in the pathogenesis of BA is NR3C1; its official name is a nuclear receptor subfamily 3, group C, member 1 (as determined by HUGO Gene Nomenclature Committee) that encodes glucocorticoid receptor (GCR). As of today, 2571 polymorphisms of this gene are known, but the most common is the Bcl-1 gene polymorphism of glucocorticoid receptor (GCR). The aim of our study was to investigate the influence of Bcl-1 gene polymorphism of glucocorticoid receptor on phenotypic expressions of BA. Materials and methods.188 patients with BA were examined within our study. We divided them into groups according to the severity of BA. The first group included 27 individuals with mild persistent BA, the second group consisted of 98 patients with moderate persistent course and 63 people with severe persistent BA constituted the third group. They were diagnosed on the basis of the GINA recommendations (2011) and Decree ¹128 of the Ministry of Health. All patients were treated according to the severity of disease. DNA was extracted from the whole blood white cells by means of using DIAtom DNA Prep 100 device («Isogene», Russia). The determination of allelic polymorphism in the second exon of the GCR gene, Bcl1 (C647G) - rs41423247, was performed by polymerase chain reaction, followed by subsequent restriction fragment length analysis [1] with modifications. Results. Analysis of the results showed that the CC genotype prevailed among the first group patients and constituted 48.1% of the total number of patients; the CG and GG genotypes were approximately equally distributed among the rest of the individuals and constituted 29.6% and 22.2%, respectively. Most of the patients with moderate persistent BA had CG genotype (46.9%). 30.6% of individuals had GG genotype and 22,4% had ÑÑ genotype. In the third group, GG genotype was observed in 46%, ÑG genotype -- in 41.3% and ÑÑ genotype -- in 12.7% of patients. Conclusions. GG genotype of Bcl-1 gene polymorphism of glucocorticoid receptor is associated with BA severity and is more likely to be present in the individuals with severe persistent BA course.
